# Malignant Phosphaturic Mesenchymal Tumor With Lung Metastasis

**DOI:** 10.7759/cureus.102504

**Published:** 2026-01-28

**Authors:** Seden Arsoy Sahin, Cem Comunoglu, Sevda Karyagar, Tanju Berber, Osman Emre Aycan

**Affiliations:** 1 Pathology, Prof. Dr. Cemil Taşcıoğlu City Hospital, Istanbul, TUR; 2 Nuclear Medicine, Prof. Dr. Cemil Taşcıoğlu City Hospital, Istanbul, TUR; 3 Radiation Oncology, Prof. Dr. Cemil Taşcıoğlu City Hospital, Istanbul, TUR; 4 Orthopaedic Oncology, Baltalimani Bone Diseases Training and Research Hospital, Istanbul, TUR

**Keywords:** case report, fgfr1, fn1, malignant, phosphaturic mesenchymal tumor

## Abstract

Malignant phosphaturic mesenchymal tumors (MPMT) are rarely seen soft tissue tumors. They can result in tumor-induced osteomalacia with hypophosphatemia. These tumors show FN1::FGFR1/FGF1 gene fusions. We present a 59-year-old male patient with a swelling in his right knee. Magnetic resonance imaging examination revealed a soft tissue mass with a maximum diameter of 2 cm in his distal right thigh. Histopathologically, the tumor was composed of atypical spindle cells. Coagulative tumor cell necrosis, extensive osteoid-like matrix, calcifications, and aneurysmal bone cyst-like areas were present. Mitotic index was 16/mm^2^. The patient had a low blood phosphorus level, a high alkaline phosphatase level, and a normal calcium level. FN1::FGFR1 fusion was detected by next-generation sequencing (NGS) method. A diagnosis of MPMT was made. Twenty months after the initial diagnosis, newly developed nodules in the lungs were detected by the PET-CT scan. Temozolomide administration was initiated. We report a patient with an MPMT who presented to the clinic with soft tissue swelling as the first complaint. The NGS method contributed to the diagnostic processes. After 35 months of follow-up, he is alive with lung metastasis.

## Introduction

Phosphaturic mesenchymal tumors (PMTs) are extremely rare. They constitute approximately <0.01 of soft tissue tumors [1,2]. They mostly occur in middle-aged patients with equal involvement of both sexes [2]. They can occur in the bone or in the soft tissues. The malignant form has been reported much less frequently. PMTs are a rare cause of tumor-induced osteomalacia (TIO), a paraneoplastic syndrome that results in renal phosphate loss and decreased bone mineralization [1,3]. The secretion of fibroblast growth factor 23 (FGF23) by the tumor plays a role in this process [4]. Most patients have a long history of fatigue or neuromuscular symptoms caused by osteomalacia, and they usually present to the clinic with complaints related to chronic hypophosphatemia [2]. These tumors exhibit FN1::FGFR1/FGF1 gene fusions [4]. Pathological diagnosis is extremely difficult because microscopic findings and immunohistochemical features of the tumor are not specific [1-3]. There are some difficulties in achieving success with laboratory and molecular diagnostic methods [4]. Malignant tumors usually show local recurrence and metastasize to the lungs [2]. Complete surgical resection is curable for PMTs; however, currently, there is no effective therapy for the malignant forms [1,2]. In this report, a malignant phosphaturic mesenchymal tumor (MPMT) of the soft tissues containing FN1::FGFR1 fusion will be presented.

## Case presentation

**Figure 1:**
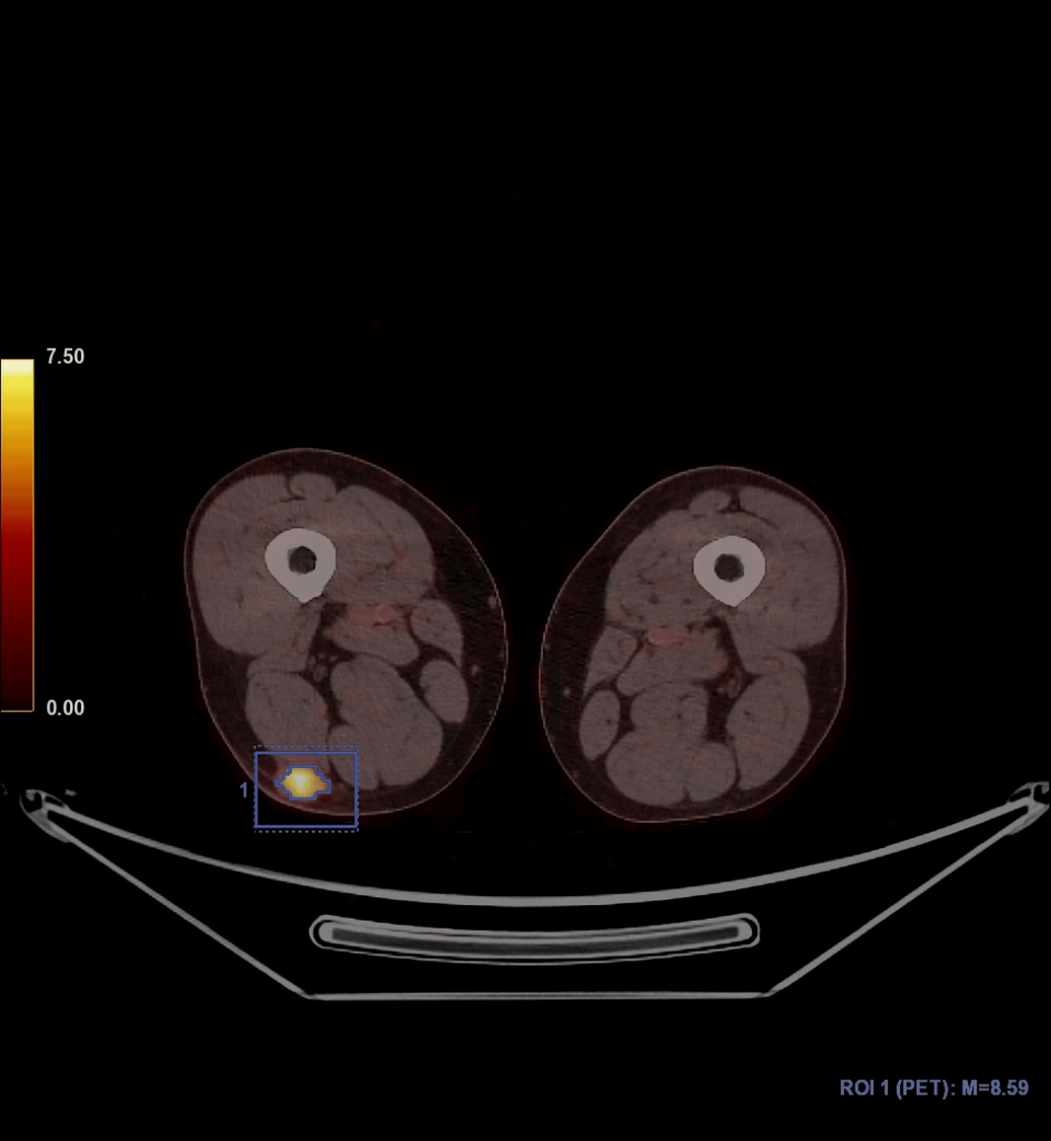
Mass lesion on PET-CT Soft tissue mass lesion located at right thigh displaying high FDG uptake (within square) (PET-CT).

**Figure 2:**
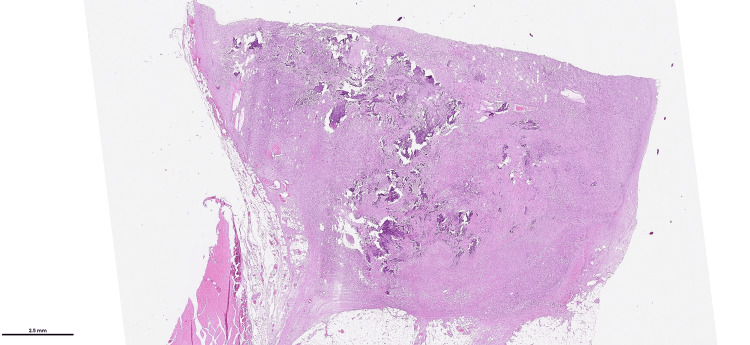
Low-power view of the tumor Low-power view of the tumor having slightly irregular borders. The prominent matrix production of so-called “grungy” calcification is readily seen at low power view (H&E, ×20).

A 59-year-old male patient was admitted to the hospital due to swelling of his right knee. He did not have bone pain. He had no complaints of muscle weakness or any functional limitations. A magnetic resonance imaging examination revealed a soft tissue mass with a maximum diameter of 2 cm in the distal posterior part of the right thigh. Laboratory findings included low blood phosphorus level (1.57 mg/dL (range, 2.5-4.5 mg/dL)), high alkaline phosphatase level (256 U/I (range, 40-129 U/I)), and normal calcium level (9.32 mg/dL (range, 8.6-10.2 mg/dL)). A PET-CT scan revealed a subcutaneous hypermetabolic mass lesion (SUVmax: 8.59) consistent with a primary malignant tumor (Figure 1) and widespread lytic bone lesions, including those in the anterior region of the right acetabulum, the bilateral iliac bones, the lateral condyle of the left femur, the posterolateral region of the left 11th rib, the right pedicle of the L3 vertebra, the vertebral body of the L1 vertebra, the calvarium, and the collum of the right femur. There were no bone fractures. A Jamshidi needle (Becton, Dickinson and Company, Franklin Lakes, NJ) biopsy of the lytic lesion at the lateral condyle of the left femur showed no tumoral lesion. The histopathological examination of the excisional biopsy material of the soft tissue mass revealed a neoplastic hypercellular spindle cell lesion forming a fascicular pattern, interspersed with notable so-called “grungy” and chicken-wire-like calcification (Figure 2).

**Figure 3:**
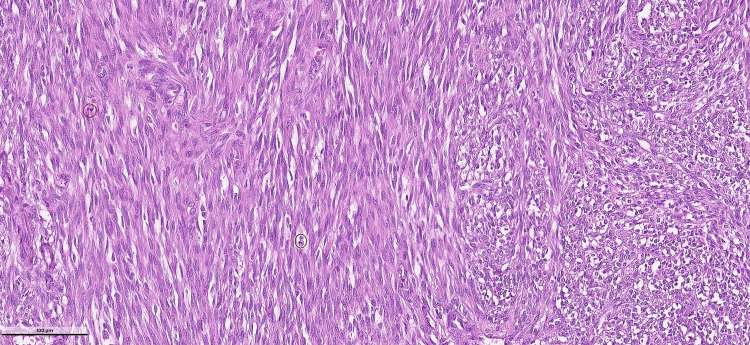
Atypical spindle cells Neoplastic spindle cells displaying cellularity and atypia are forming a fascicular pattern; mitotic figures are observed (within the circles) (H&E stain, ×400).

The spindle cells displayed cellular atypia (Figure 3).

**Figure 4:**
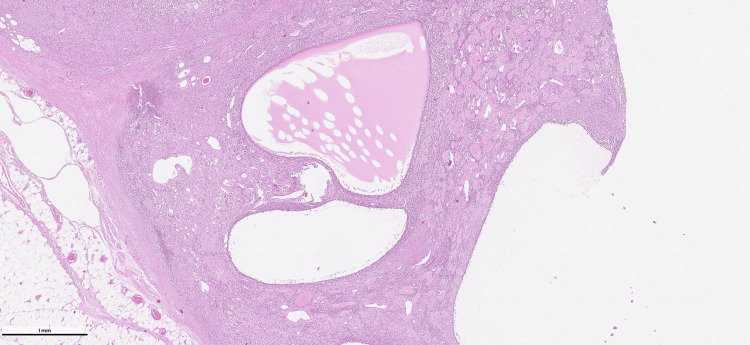
Aneurysmal bone cyst-like areas Multiple aneurysmal bone cyst-like areas (H&E, ×10).

Areas of coagulative tumor cell necrosis were present. The mitotic figures were 16/mm² at their highest. Extensive osteoid-like matrix, areas of calcification, microcystic change, and aneurysmal bone cyst-like structures were observed (Figure 4).

**Figure 5:**
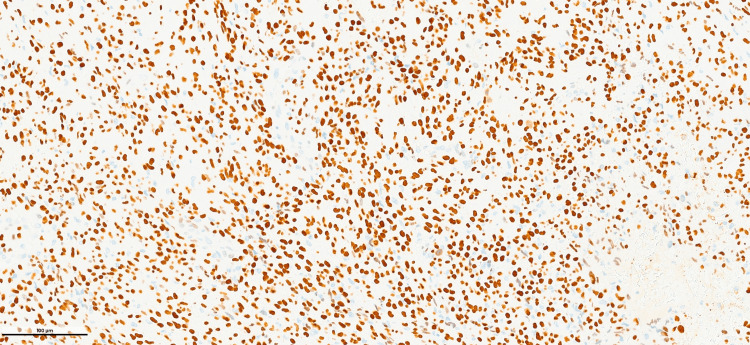
SATB2 immunopositivity SATB2 is immunopositive in tumor cells (SATB2; ×200).

The borders of the tumor were slightly irregular, but the surgical margins were free from tumor. Immunohistochemically, only SATB2 positivity was observed (Figure 5).

**Figure 6:**
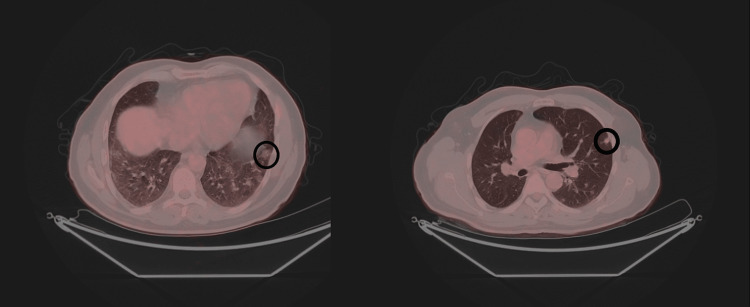
Metastatic nodules in the lung Metastatic nodules, the largest measured as 12 mm, are seen in the lung (within the circles) (PET-CT).

Keratins, p40, S100, desmin, smooth muscle actin, CD34, ERG, TLE1, and CD99 were immunonegative. The Ki-67 proliferation index was 15-20%. A high-grade malignant mesenchymal tumor was considered, and extraskeletal osteosarcoma and MPMT were included in the differential diagnosis. The next-generation sequencing (NGS) method was applied, and the FN1::FGFR1 fusion was detected. A diagnosis of MPMT was given. Three months after the excisional biopsy, blood phosphorus levels were within normal levels. Twenty months after the initial diagnosis, a PET-CT scan showed newly developed nodules exhibiting irregular borders in both lobes of the lungs. These nodules were microlobulated with spiculated contours. The largest nodule measured 9 mm in maximum diameter (SUVmax: 2.35). A lung biopsy was not feasible. There was no recurrent lesion present at the primary site. Since these nodules were absent during the patients' initial consultation, they were deemed metastatic foci. Temozolomide administration (100 mg twice per day) was initiated. During the patient’s most recent visit (27 months after the initial diagnosis), the nodules appeared to increase in size and number. The largest nodule measured 12 mm in maximum diameter (SUVmax: 6.00) (Figure 6).

During his last control visit, his bone lesions were stable, and his blood phosphorus level was low. We have presented the changes in the blood levels of phosphorus and alkaline phosphatase, calcium, and parathormone in Table 1.

**Table 1:** Serum levels of alkaline phosphatase and phosphorus during the course of the disease ALP: alkaline phosphatase; Ca: calcium; P: phosphorus; PTH: parathormone

	ALP (U/I) (40-129)	P (mg/dL) (2.5-4.5)	Ca (mg/dL) (15-88)	PTH (pg/mL) (8.6-10.2)
Before surgery	256	1.57	98.3	9.82
After surgery	101	3.26	47.5	9.93
At recurrence	171.65	1.3	102.3	9.14

The patient is alive with the disease 35 months after the initial diagnosis, and treatment continues.

## Discussion

The patients diagnosed with PMT are typically reported to present to the clinic with pathological fractures in the setting of chronic hypophosphatemic, hyperphosphaturic osteomalacia, as well as gradual muscle weakness, bone pain, and difficulty walking [3]. Clinical symptoms often begin several months before the diagnosis of PMT is given [5,6]. Alkaline phosphatase levels are generally found to be elevated in PMT patients [5]. High serum levels of FGF23 help with the diagnostic process [6]; however, we were unable to measure it. Although patients with PMT usually present to the clinic with symptoms related to TIO, at his first clinic visit, he had no complaints other than swelling in his right knee. In our patient, alkaline phosphatase levels were high, and hypophosphatemia was present.

Tumors apart from PMT have also been reported to cause TIO [1]. However, despite their broad histopathological spectrum, these tumors have been reported to represent a distinct neoplasm, PMT [1,3].

Although the criteria for malignancy in PMT are not clearly defined, nuclear pleomorphism, increased cellularity, increased mitotic activity, and necrosis have been mentioned to indicate malignancy [2]. Folpe et al. [1] found that mitotic activity exceeded 5/10 HPF in malignant PMT. It has been reported that a preexisting benign component of PMT was found in malignant PMT lesions [6]. Despite a meticulous macroscopic sampling, we could not find a benign component within our tumor. Folpe [6] claimed that matrix production was not associated with MPMT. Our tumor showed an extensive osteoid-like and fibrotic matrix.

FN1::FGFR1 or FN1::FGF1 fusions have been reported to be detected in most PMTs. Lee et al. claimed that they could detect FN1::FGFR1 fusion by FISH analysis in nine of their 15 patients (60%) [4]. However, Yamada et al. [7] reported finding this event in only two of their 17 tumors by the FISH method (12%). Lee et al. detected FN1::FGFR1 fusion in three of four tumors using RNA sequencing NGS (75%) [4]. We think NGS may be an alternative diagnostic method.

Histopathological diagnosis is important in order to administer the correct oncological treatment. The differential diagnosis especially includes extraskeletal osteosarcoma [6]. Osteoid-like matrix production and immunohistochemical SATB2 positivity, as observed in our tumor, make the distinction between PMT and extraskeletal osteosarcoma even more difficult. Matrix production in the form of so-called “grungy” calcification is characteristic of these tumors [6]. However, chicken-wire-like calcification is not a specific finding and has not been reported before to the best of our knowledge.

It has been claimed that in malignant and/or inoperable PMTs, FGFR antagonists could be used [4]. Burosumab is a monoclonal antibody against FGF23. It has been claimed that burosumab therapy improves osteomalacia and corrects phosphate metabolism [8,9]. Because it does not treat the disease, it can be an option for patients with an unresectable tumor [8,9]. Infigratinib is an FGFR1-3 tyrosine kinase inhibitor. A partial positive response was observed with infigratinib treatment; however, severe adverse effects such as ocular scarring were reported [8,9]. Although there are hopes for the development of novel therapeutics, there is, unfortunately, currently no effective treatment agent. Therefore, for our patients’ treatment, we had to choose temozolomide. It has been claimed that this agent had limited efficacy against sarcomas [10]. Unfortunately, newly formed metastatic nodules were observed in our patient under temozolomide treatment.

## Conclusions

As a conclusion, we are presenting an MPMT case metastasized to the lung without local recurrence. The patient presented with a soft tissue mass in his right knee. During his visit, his serum phosphorus levels were low, and alkaline phosphatase levels were high. Radiological examinations revealed multiple osteolytic lesions. After the diagnosis of PMT and total resection of the tumor, serum phosphorus and alkaline phosphatase returned to normal levels. Twenty months after the surgery, he had multiple lung metastases. Histopathologically, against the major observance, the tumor exhibited prominent matrix production, including chicken-wire-like calcification that had not previously been documented. Follow-up of the patient is continuing, and he is alive 35 months after the initial diagnosis. MPMT is a special and rare tumor in that it requires clinical information and histopathology to be evaluated together. We think the NGS method can also be used as an auxiliary diagnostic tool.
